# Acute coronary syndrome as the initial manifestation of infective endocarditis in an apparently normal native valve: a case report

**DOI:** 10.1093/ehjcr/ytae593

**Published:** 2024-11-06

**Authors:** Felipe Lozano Pineda, Alberto Navarro Navajas, Juan Manuel Senior

**Affiliations:** Clinical Cardiology, The University of Antioquia, The San Vicente University Hospital Foundation, 67th Street # 53-108, 050010 Medellín, Colombia; Interventional Cardiology, The University of Antioquia, The San Vicente University Hospital Foundation, 67th Street # 53-108, 050010 Medellín, Colombia; Interventional Cardiology, Cardiology Section, Department of Internal Medicine, Cardiovascular Disease Study Group, The University of Antioquia, Hemodynamics Service, Integrated Cardiopulmonary and Peripheral Vascular Functional Unit, The San Vicente University Hospital Foundation, 67th Street # 53-108, 050010 Medellín, Colombia

**Keywords:** Endocarditis, Bicuspid aortic valve, Acute coronary syndrome, Cardiac tomography, Case report

## Abstract

**Background:**

Acute coronary syndrome is a rare complication of infectious endocarditis. We present the case of a patient with a bicuspid aortic valve who presented an acute ST elevation myocardial infarction (STEMI) of the inferior wall secondary to vegetation that generated obstruction of the ostium of the right coronary artery (RCA).

**Case summary:**

A 54-year-old patient with only a history of smoking was admitted for chest pain. An acute STEMI in the inferior wall was documented; he underwent an emergent coronary angiography, which showed a mass that obstructed the ostium of the RCA that did not allow its channelling. An angiotomography of the aorta was performed, where dissection was ruled out and the presence of a mass in the right coronary sinus that protruded towards the ostium of the coronary artery was confirmed. In the extension studies, a bicuspid aortic valve and thrombosis in the right brachial artery and in the infrarenal abdominal aorta were documented.

**Discussion:**

The case was taken to a medical meeting and given the embolic and mechanical compromise; it was decided to perform surgery where a mass that invaded the proximal segment of the RCA was resected. The histopathological study documented findings of vegetation; the cultures were positive for *Staphylococcus epidermidis*, and he finally received antibiotic and anticoagulation treatment.

**Learning points**

- To understand that acute myocardial infarction is a complication of infective endocarditis.
- To recognize subtle cardiac alterations such as the bicuspid aortic valve that can predispose to infectious processes.
- To recognize that multiple systemic embolic phenomena can support the presence of infective endocarditis.

## Introduction

Infectious endocarditis (IE) is a disease with high mortality and embolic events. Acute coronary syndrome (ACS) is a rare complication in the context of IE (2.9%), but it is associated with a higher risk of cardiovascular complications, such as heart failure (29%) and in-hospital mortality (14%).^1,2^ Although having underlying structural heart disease or having undergone previous surgery are important risk factors for the development of IE, up to 40% of cases have no history of heart disease. We present the case of an unusual clinical manifestation of IE debuting with myocardial infarction in a patient with a bicuspid aortic valve.^3.4^

## Summary figure

**Table:** 

Timeline	
Day 0	Emergency room visit due to oppressive chest pain; inferior wall ST infarction was documented; an emergent coronary arteriography was performed without being able to cannulate the right coronary artery due to possible mass confirmed on tomography.
Day 1	Documentation of acute ischaemia of the lower limbs and thrombosis of the right brachial artery and infrarenal aorta.
Day 2	Emergent surgery for resection of mass in the right sinus of Valsalva, detection of a bicuspid aortic valve (at surgery), infrapatellar and right brachial artery thromboembolectomy was performed, admission to Intensive Care Unit.
Day 5	Right heart failure requiring inotropy and vasopressor.
Day 10	Histopathology compatible with vegetation.
Day 16	Antibiotic continuity and anticoagulation titration with warfarin.
Day 20	Discharge with outpatient antibiotics (vancomycin) and anticoagulation.
Day 50	Follow-up. He returns to work without consequences.

### Case presentation

A 54-year-old male patient presented oppressive chest pain, which began at rest and was very intense from the beginning, associated with dyspnoea and radiation to the right upper limb. He did not show fever and had not undergone any recent surgeries or interventions. His blood pressure upon admission was 190/110, and he had weak pulses in his lower limbs; he had no other alterations relevant to the physical examination. His background was that of an active smoker, with a pack-year index of 21. He had no relevant pathological or surgical history, nor did he take chronic medication.

**Figure 1:**
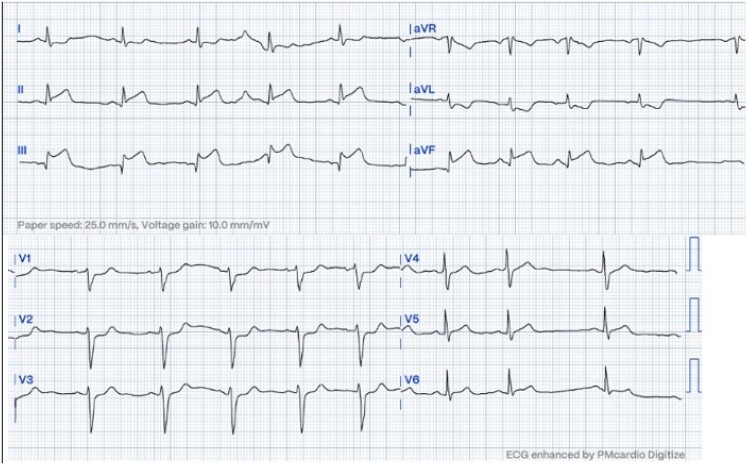
The electrocardiogram (ECG) showed ST segment elevation in the inferior leads and ST segment depression in DI and aVL.

**Figure 2:**
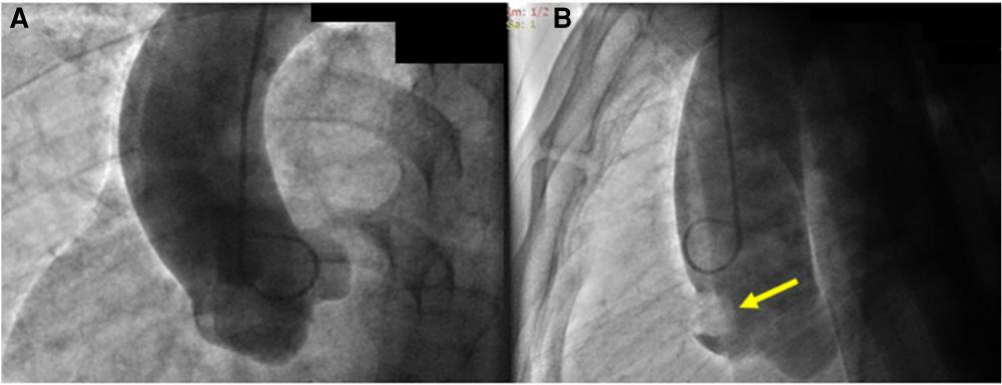
(*A*) Aortogram where the right coronary artery (RCA) is not visualized. (*B*) Mass obstructing the ostium of the RCA (arrow).

**Figure 3:**
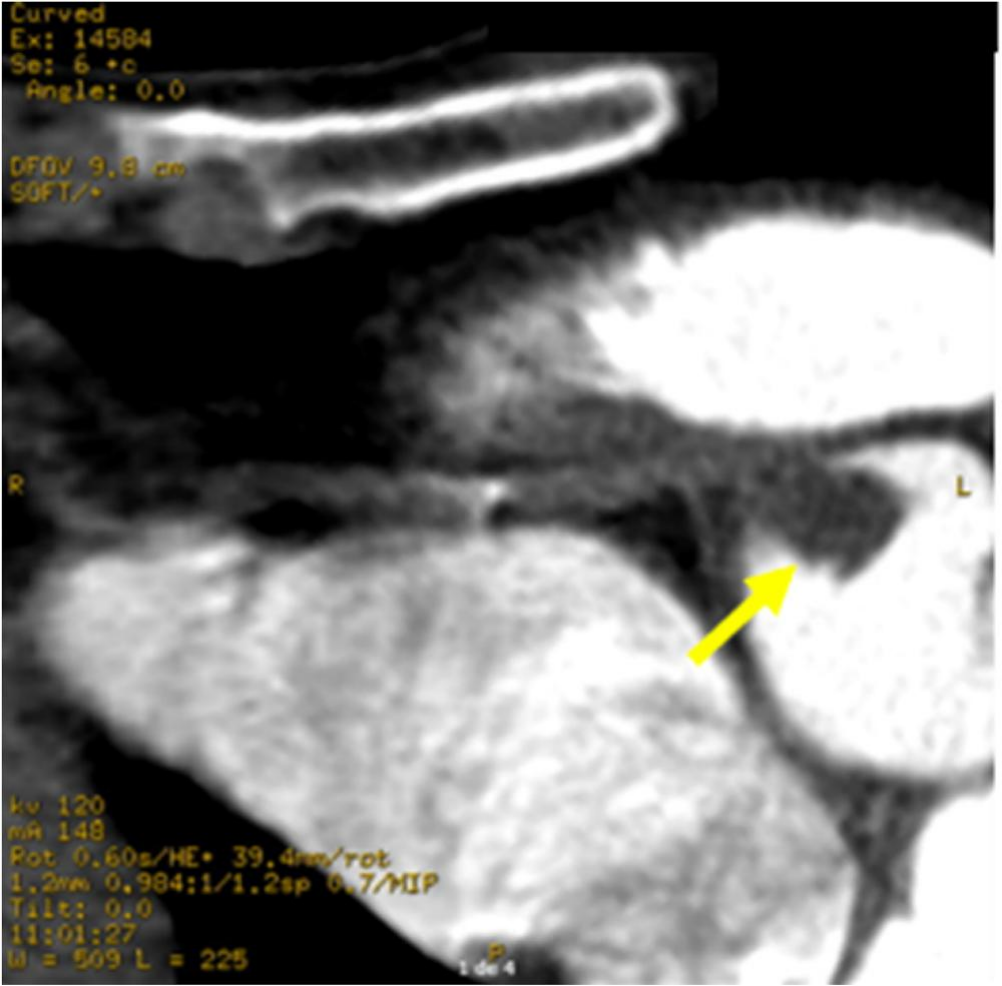
Angiotomography of the ascending aorta shows obstruction of the RCA ostium due to a mass (yellow arrow).

Upon his admission to the Emergency Department, an electrocardiogram was performed, which showed ST segment elevation in the inferior leads with reciprocal changes in the upper lateral wall suggestive of right coronary artery (RCA) involvement (*Figure 1*). He was taken to an emergent coronary arteriography where it was evidenced that the left system was free of significant disease (see Supplementary material online, *Video S1*). Since the ostium of the RCA could not be cannulated, it was decided to perform an aortogram where the presence of a mass or occlusive thrombus in the ostium of the RCA was evidenced (*Figure 2* and Supplementary material online, *Videos S2* and *S3*). An angiotomography of the aorta was performed, which ruled out dissection and documented the presence of a mass in the right coronary sinus causing obstruction of the ostium of the RCA (*Figure 3*), in addition to a filling defect due to the presence of a thrombus in the infrarenal abdominal aorta (see Supplementary material online, *Figure S1*). The differential diagnoses that were considered at the beginning included a dissection of the ascending aorta with involvement of the right ostium, an obstructive thrombus of a cardioembolic source, IE, and ACS due to atherosclerotic disease of the ostium of the RCA. The initial treatment consisted of anti-ischaemic management (given the suspicion of ACS), anticoagulation, and finally, empirical antibiotics.

**Figure 4:**
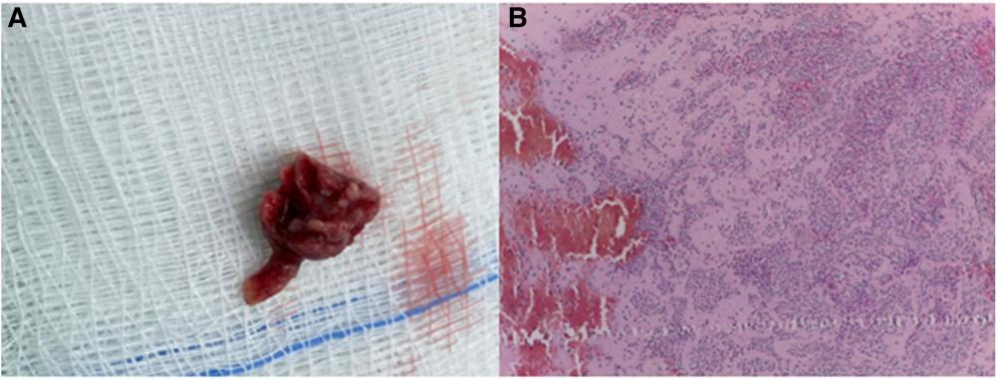
(*A*) Mass in the right coronary sinus with a portion in the proximal segment of the RCA. (*B*) Tissue stained with haematoxylin and eosin. Multiple irregular fragments of dense connective tissue, fibrin accumulations, haemorrhagic areas, and dense polymorphonuclear aggregate neutrophils with areas of necrosis are found.

Given the clinical involvement and evidence of peripheral arterial thrombosis (infrarenal and right brachial artery), it was decided to perform emergent surgery, where a 2.5 cm mass was observed that was above the sinus of the right coronary leaflet that invaded the proximal segment of the RCA in a bicuspid aortic valve (*Figure 4*). With these findings, resection of the mass and a thromboembolectomy of the RCA were performed. At that same time, due to suspicion of acute ischaemia of the lower extremities, an infrapatellar and right brachial artery thromboembolectomy was performed. Post-operatively, he developed right heart failure that required temporary vasopressor support. The biopsy of the mass showed histopathological findings suggestive of vegetation (*Figure 4*) and in the cultures of the mass, *Staphylococcus epidermidis* was isolated; the peripheral blood cultures were negative, and no immunosuppression was detected. The patient was determined to have definite IE (by meeting one major and three minor criteria).

During follow-up, an echocardiogram was performed at 3 months, which showed no serious structural alterations (except for the presence of bicuspid aortic valve) (see Supplementary material online, *Video S4*). The patient was discharged as there were no complications. It was decided to treat him with intravenous vancomycin for three weeks, carvedilol, enalapril, and anticoagulation with warfarin.

## Discussion

The case of a patient with acute myocardial infarction as the first manifestation of IE associated with a bicuspid aortic valve without inflammatory response at presentation is described. Due to the presence of multiple thrombi in different territories, it was necessary to rule out an infectious phenomenon that led, in this case, to be taken to surgery to have the correct diagnosis.

A rare situation is the presence of IE in valves that morphologically appear normal (26%, in some records). However, some cohorts have shown that mitral valve prolapse, and the bicuspid aortic valve, is the most common in IE cases with apparently normal valves.^4^

Acute myocardial infarction may be the first manifestation of IE. The two main mechanisms that explain this phenomenon are coronary embolism and extrinsic compression due to periannular complications. Many IE cases presenting an acute coronary event in observational studies do not show atherosclerotic coronary disease.^5^

Multimodal imaging is essential for diagnosis, since, in complex cases, several complementary strategies are required. Management in these cases is a challenge since infectious processes can predispose to haemorrhagic complications with the use of thrombolytics or to perpetuation of the infection with percutaneous interventions (formation of mycotic aneurysms).^6^ In this specific case, surgery was chosen due to the patient’s mechanical and embolic compromise, a decision that, depending on each case, requires a multidisciplinary approach.

## Conclusions

Acute coronary syndrome as the initial manifestation of IE is rare. It is important to keep in mind that there are subtle structural lesions, such as the bicuspid aortic valve, that may be associated with the appearance of IE. The presence of multiple embolic phenomena should always raise suspicion of an underlying infectious cause.

## Lead author biography


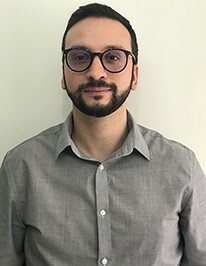


Felipe Lozano is an internist; he is currently a clinical cardiology fellow at the University of Antioquia. His main interest is in topics related to heart failure.

## Supplementary Material

ytae593_Supplementary_Data

## Data Availability

The data underlying this article are available in the article and in its online Supplementary material.

## Notes

**Consent:** The authors confirm that written consent has been obtained from the patient for the presentation and publication of this case report, including images and associated text, in accordance with the Committee on Publication Ethics (COPE) guidelines.

**Funding:** The authors have not received funding.
