# Supplementary material for: Acute coronary syndrome as the initial manifestation of infective endocarditis in an apparently normal native valve: a case report
Source: Eur Heart J Case Rep. 2024 Nov 6;8(11):ytae593. doi: 10.1093/ehjcr/ytae593 (PMC11552525; doi:10.1093/ehjcr/ytae593)
Supplement: ytae593_Supplementary_Data [file ytae593_supplementary_data.zip › Supplement Figure 1.docx]

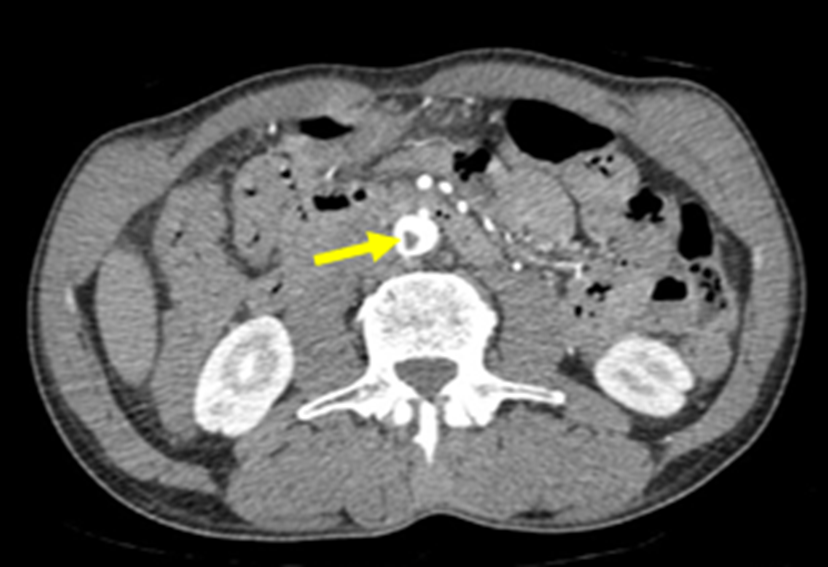


**Supplementary Figure 1:** Angiotomography of the abdominal aorta. Presence of filling defect (thrombus) in the infrarenal aorta (yellow arrow).
